# Reinventing Cellulose into Sustainable and Versatile Bioplastics via Molecular-Scale Design

**DOI:** 10.34133/research.1289

**Published:** 2026-05-13

**Authors:** Minxin Wang, Geyuan Jiang, Suqing Zeng, Dawei Zhao, Haipeng Yu

**Affiliations:** ^1^ Key Laboratory on Resources Chemicals and Materials of Ministry of Education, Shenyang University of Chemical Technology, Shenyang 110142, P. R. China.; ^2^ State Key Laboratory of Woody Oil Resources Utilization, Northeast Forestry University, Harbin 150040, P. R. China.

## Abstract

Cellulose, the most abundant natural polymer, is a promising platform for sustainable bioplastics. However, cellulose-based bioplastics derived from macrofibers or nanocellulose still fall short of petrochemical plastics in thermal stability, mechanical performance, and processability. In this work, we systematically examine molecular-scale design strategies to bridge the gap between material properties and processing behavior, including supramolecular network reconstruction, dynamic dissipative systems, and programmable architectures. These approaches enable cellulose bioplastics with improved formability, thermal resistance, mechanical strength, and programmable responsiveness, thereby expanding their potential in aerospace, intelligent construction, high-end protective equipment, and biomedicine. Finally, we outline future research directions to accelerate the development of cellulose-based materials toward high-performance, recyclable, intelligent, and environmentally friendly applications.

Traditional petrochemical plastics suffer from nondegradability, microplastic pollution, and high carbon emissions, hindering sustainable development (Fig. 1). Bioplastics with renewability and carbon-neutral potential can form a closed carbon cycle, serving as promising alternatives to petroleum-based plastics (Fig. 1). Nevertheless, mainstream bioplastics (such as polylactic acid, polyhydroxyalkanoate, etc.) are limited by poor mechanical performance and uncontrollable degradation, restricting their large-scale substitution [1].

**Fig. 1.**
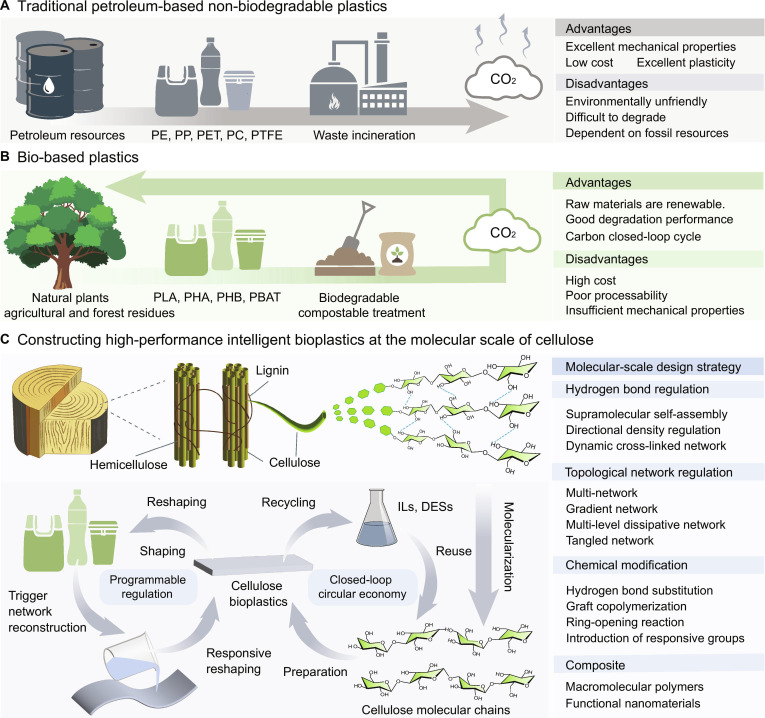
Traditional petrochemical plastics and bioplastics. (A) The life cycle, advantages, and disadvantages of traditional petrochemical plastics and (B) cellulose bioplastics. (C) Constructing closed-loop recycling bioplastics at the molecular scale of cellulose.

As a renewable polymer material with wide sources, abundant reserves, low cost, and complete biodegradability, cellulose is an ideal raw material for the preparation of bioplastics. However, in the traditional synthesis process of bioplastics, cellulose is mainly used in the form of macro-sized fibers, or nanocellulose is adopted as a plastic filler. During the production and processing of such materials, issues including uneven fiber orientation, poor interfacial compatibility, insufficient dispersibility, and unsatisfactory melt fluidity often restrict their thermal, mechanical, and processing properties. These issues have severely restricted the application of bioplastics in replacing traditional petrochemical plastics, especially in high-end engineering fields [2]. To address these drawbacks, precise regulation at the molecular level, including dynamic hydrogen bond regulation, network topology reconstruction [3], and multiscale regulation [4], the precise coupling of comprehensive properties such as strength, toughness, thermal stability, and programmability of bioplastics can be achieved (Fig. 1) [5,6]. Such performance advancement greatly broadens the application scope of cellulose bioplastics, extending from conventional packaging to high-value fields including aerospace, intelligent architecture, advanced protection, and biomedicine.

The macroscopic properties of materials are essentially determined by the microscopic molecular and supramolecular network structures. Molecularization can effectively destroy the natural multiscale structure and hydrogen bond network of cellulose, form a series of controllable molecular chains or functionalized monomers, transform the rigid and ordered cellulose network into a designable dynamic network, and thus realize the precise regulation of the macroscopic properties of materials [7,8]. Thermal processability is a prerequisite for the large-scale production of plastics. However, the crystalline regions and highly ordered hydrogen bond networks inside cellulose severely restrict the mobility of molecular chains. As a result, molecular chains undergo thermal decomposition at high temperatures before they gain sufficient mobility [9]. To solve this problem, dynamic imine bonds can be introduced into the hydrogen bond network to weaken the strong hydrogen bonding interaction between cellulose molecular chains. Meanwhile, the thermally triggered dynamic exchange characteristic of imine bonds is utilized to enhance the mobility of polymer chain segments, realize stress relaxation of the cellulose plastic network, and markedly improve the thermal processability of the material. Meanwhile, by regulating the arrangement and aggregation structure of cellulose molecular chains, the crystallization process can be induced and promoted, which facilitates the formation of crystalline regions with more complete structures and denser arrangements. This effectively inhibits the thermal movement and thermal degradation of molecular chains and improves thermal stability [10,11].

At present, cellulose bioplastics generally suffer from high brittleness and mechanical properties far inferior to those of petroleum-based plastics. In recent years, the research direction of bioplastics has gradually shifted from pursuing “passive enhancement” based on covalent cross-linking to designing controllable dynamic energy dissipation systems [12,13]. The essence of energy dissipation does not stem from the absolute rigidity of the material to resist deformation, but lies in the controllable, multiscale, and reversible dissociation and recombination processes of its internal structure under stress. Such systems include (a) the breaking and recombination of reversible bonds (hydrogen bond networks) and (b) entanglement and chain slippage. Under external forces, the breakage of reversible bonds is preferentially triggered to dissipate stress, effectively preventing brittle fracture. When the external force weakens, the reversible bonds recombine to maintain structural stability, enabling the material to dissipate energy while retaining excellent structural strength and stability. In addition, grafting flexible side chains or inducing structural reconstruction can promote the interpenetration and entanglement of molecular chains to form an entangled structure, which features dynamic reversibility and restricted segment movement. Under external force, the chain segments between entanglement points undergo stretching, slipping, and disentanglement to consume energy. The combination of the 2 strategies can construct a network structure with both dynamic slipping characteristics and structural stability [14], making it suitable for high-end application scenarios with high bearing capacity and high stress, such as lightweight aerospace components and engineering construction fields.

Through precise molecular-scale design, sensitive functional groups responsive to specific external stimuli such as heat, light, and water are pre-encoded into the cellulose network. This enables the reversible reorganization of the internal structure of materials at different stages, including processing, service, and recycling merely by applying corresponding stimuli [3,15,16]. Meanwhile, this process only involves the reconstruction of network topological structures without damaging molecular main chains and functional units. Accordingly, the recycled materials maintain a higher retention rate of properties, which effectively avoids issues such as downcycling [17,18]. As a natural hydrophilic polymer, cellulose can achieve dynamic remodeling relying on its inherent water-responsive properties. Through water-induced dynamic dissociation and recombination of molecular networks, materials can undergo reversible switching between the “rigid stable state” and the “flexible plastic state” [3,19,20]. Although this strategy avoids problems such as high-temperature thermal processing and difficult recycling, in high-humidity environments, plasticization and deformation caused by moisture will lead to a series of issues including poor dimensional stability and uncontrollable mechanical properties of materials.

In summary, by regulating cellulose at the molecular level, the inherent defects of cellulose itself can be effectively compensated, further promoting the development of cellulose into high-performance, recyclable, and intelligent materials. The future research directions are as follows: (a) Achieve high-level properties like extreme stability, self-healing, and multifunctionality to expand high-end application scenarios. (b) Make full use of artificial intelligence and machine learning to guide the reverse design of materials. (c) Optimize life cycle environmental properties to realize the green and sustainable circulation of cellulose bioplastics. (d) Reduce production costs through low-cost treatment and cyclic utilization, enhancing the practical competitiveness of cellulose-based materials.
